# PlateletBase: A Comprehensive Knowledgebase for Platelet Research and Disease Insights

**DOI:** 10.1093/gpbjnl/qzaf031

**Published:** 2025-04-12

**Authors:** Huaichao Luo, Changchun Wu, Sisi Yu, Hanxiao Ren, Xing Yin, Ruiling Zu, Lubei Rao, Peiying Zhang, Xingmei Zhang, Ruohao Wu, Ping Leng, Kaijiong Zhang, Qi Peng, Bangrong Cao, Rui Qin, Hulin Wei, Jianlin Qiao, Shanling Xu, Qun Yi, Yang Zhang, Jian Huang, Dongsheng Wang

**Affiliations:** Department of Clinical Laboratory, Sichuan Clinical Research Center for Cancer, Sichuan Cancer Hospital & Institute, Sichuan Cancer Center, University of Electronic Science and Technology of China, Chengdu 610041, China; School of Life Science and Technology, University of Electronic Science and Technology of China, Chengdu 610054, China; Department of Medical Oncology, Sichuan Clinical Research Center for Cancer, Sichuan Cancer Hospital & Institute, Sichuan Cancer Center, University of Electronic Science and Technology of China, Chengdu 610041, China; Innovative Institute of Chinese Medicine and Pharmacy, Academy for Interdiscipline, College of Medical Technology, Chengdu University of Traditional Chinese Medicine, Chengdu 611137, China; Department of Clinical Laboratory, Sichuan Clinical Research Center for Cancer, Sichuan Cancer Hospital & Institute, Sichuan Cancer Center, University of Electronic Science and Technology of China, Chengdu 610041, China; Department of Clinical Laboratory, Sichuan Clinical Research Center for Cancer, Sichuan Cancer Hospital & Institute, Sichuan Cancer Center, University of Electronic Science and Technology of China, Chengdu 610041, China; Department of Clinical Laboratory, Sichuan Clinical Research Center for Cancer, Sichuan Cancer Hospital & Institute, Sichuan Cancer Center, University of Electronic Science and Technology of China, Chengdu 610041, China; Innovative Institute of Chinese Medicine and Pharmacy, Academy for Interdiscipline, College of Medical Technology, Chengdu University of Traditional Chinese Medicine, Chengdu 611137, China; Department of Clinical Laboratory, Sichuan Clinical Research Center for Cancer, Sichuan Cancer Hospital & Institute, Sichuan Cancer Center, University of Electronic Science and Technology of China, Chengdu 610041, China; Innovative Institute of Chinese Medicine and Pharmacy, Academy for Interdiscipline, College of Medical Technology, Chengdu University of Traditional Chinese Medicine, Chengdu 611137, China; Innovative Institute of Chinese Medicine and Pharmacy, Academy for Interdiscipline, College of Medical Technology, Chengdu University of Traditional Chinese Medicine, Chengdu 611137, China; Department of Clinical Laboratory, Sichuan Clinical Research Center for Cancer, Sichuan Cancer Hospital & Institute, Sichuan Cancer Center, University of Electronic Science and Technology of China, Chengdu 610041, China; Department of Clinical Laboratory, Sichuan Clinical Research Center for Cancer, Sichuan Cancer Hospital & Institute, Sichuan Cancer Center, University of Electronic Science and Technology of China, Chengdu 610041, China; Sichuan Key Laboratory of Radiation Oncology, Sichuan Clinical Research Center for Cancer, Sichuan Cancer Hospital & Institute, Sichuan Cancer Center, University of Electronic Science and Technology of China, Chengdu 610041, China; Chengdu Boltzmann Technology Co., Ltd., Chengdu 610041 China; Chengdu Boltzmann Technology Co., Ltd., Chengdu 610041 China; Blood Diseases Institute, Xuzhou Medical University, Xuzhou 221004, China; Department of Critical Care Medicine, Sichuan Clinical Research Center for Cancer, Sichuan Cancer Hospital & Institute, Sichuan Cancer Center, University of Electronic Science and Technology of China, Chengdu 610041, China; Department of Critical Care Medicine, Sichuan Clinical Research Center for Cancer, Sichuan Cancer Hospital & Institute, Sichuan Cancer Center, University of Electronic Science and Technology of China, Chengdu 610041, China; Innovative Institute of Chinese Medicine and Pharmacy, Academy for Interdiscipline, College of Medical Technology, Chengdu University of Traditional Chinese Medicine, Chengdu 611137, China; School of Life Science and Technology, University of Electronic Science and Technology of China, Chengdu 610054, China; Department of Clinical Laboratory, Sichuan Clinical Research Center for Cancer, Sichuan Cancer Hospital & Institute, Sichuan Cancer Center, University of Electronic Science and Technology of China, Chengdu 610041, China

**Keywords:** Platelet, Database, Omics, Knowledgebase, Enrichment

## Abstract

Platelets are vital in many pathophysiological processes, yet there is a lack of a comprehensive resource dedicated specifically to platelet research. To fill this gap, we have developed PlateletBase, a knowledge base aimed at enhancing the understanding and study of platelets and related diseases. Our team retrieved information from various public databases, specifically extracting and analyzing RNA sequencing (RNA-seq) data from 3711 samples across 41 different conditions available on the National Center for Biotechnology Information (NCBI). PlateletBase offers six analytical and visualization tools, enabling users to perform gene similarity analysis, pair correlation, multi-correlation, expression ranking, clinical information association, and gene annotation for platelets. The current version of PlateletBase includes 10,278 genomic entries, 31,758 transcriptomic entries, 4869 proteomic entries, 2614 omics knowledge entries, 1833 drugs, 97 platelet resources, 438 diseases/traits, and six analysis modules. Each entry has been carefully curated and supported by experimental evidence. Additionally, PlateletBase features a user-friendly interface designed for efficient querying, manipulation, browsing, visualization, and analysis of detailed platelet protein and gene information. The case studies on gray platelet syndrome and angina pectoris demonstrate that PlateletBase is a suitable tool for identifying diagnostic biomarkers and exploring disease mechanisms, thereby advancing research in platelet functionality. PlateletBase is accessible at http://plateletbase.clinlabomics.org.cn/.

## Graphical abstract

**Graphical abstract:**
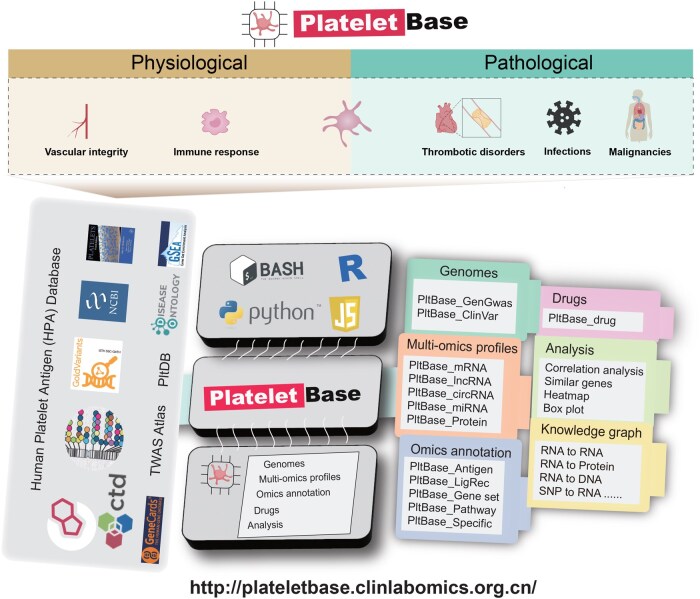

## Introduction

**Figure 1:**
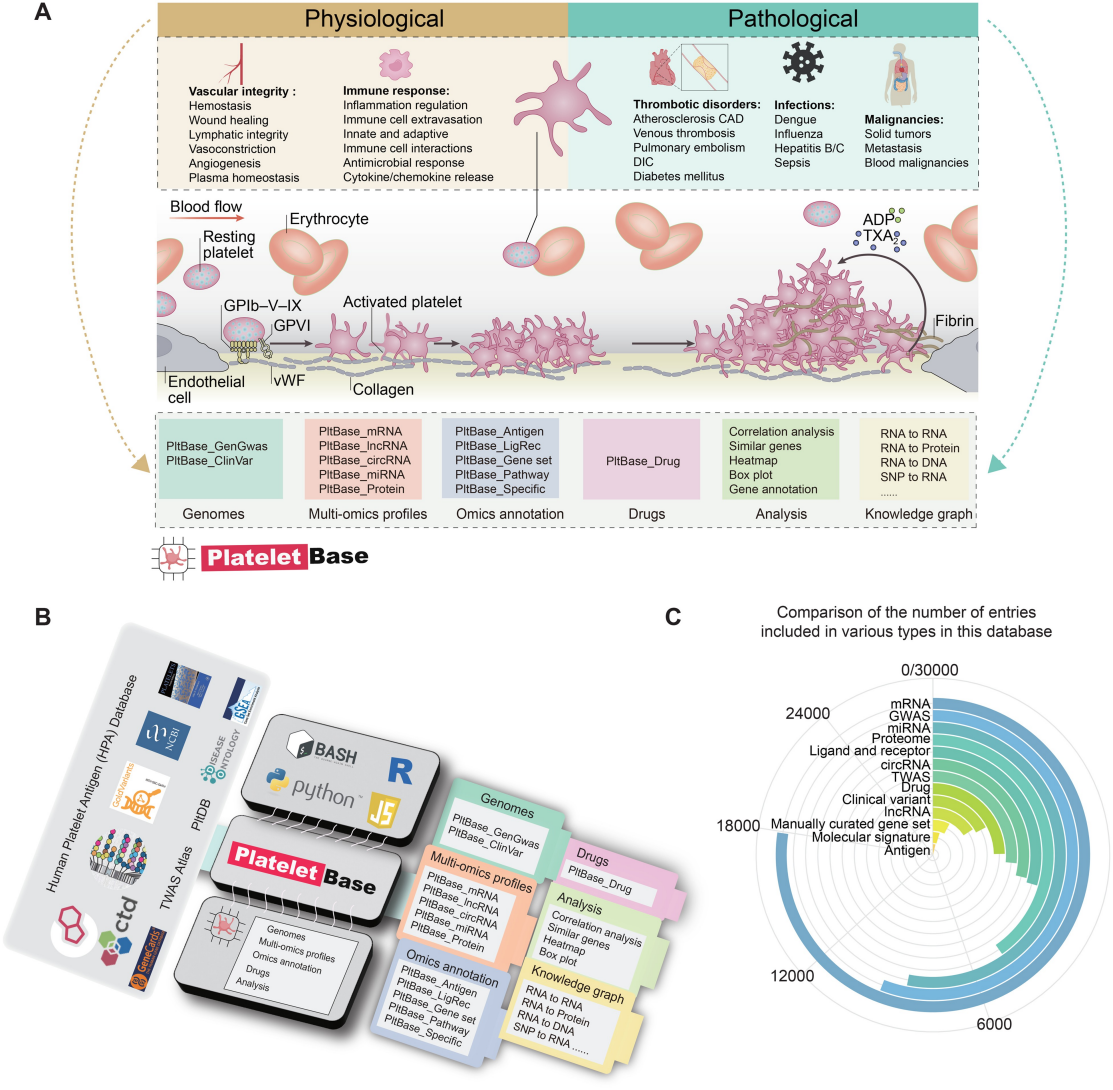
Database content and features **A**. The function of platelets in physiological and pathological processes. **B**. Schematic diagram of the database construction process. **C**. Entry statistics of various data types. CAD, coronary artery disease; DIC, disseminated intravascular coagulation; GPIb–V–IX, glycoprotein Ib–V–IX complex; vWF, von Willebrand factor; ADP, adenosine diphosphate; TXA2, thromboxane A2; mRNA, messenger RNA; miRNA, microRNA; lncRNA, long non-coding RNA; circRNA, circular RNA; GWAS, genome-wide association study; TWAS, transcriptome-wide association study; LigRec, ligand and receptor; SNP, single nucleotide polymorphism.

Platelets, which are anucleate cellular components, are generated by megakaryocytes in the bone marrow and lungs [1]. The primary function of platelets in the body is predominantly associated with the maintenance of normal blood flow through a process known as hemostasis, which plays a crucial role in preventing excessive bleeding or hemorrhaging within blood vessels [2]. Over the past decade, numerous studies have elucidated the emerging roles of platelets beyond hemostasis and thrombosis [3]. Physiologically, extensive molecular and functional research has unveiled the multifaceted roles of platelets in preserving vascular integrity, facilitating vascular remodeling, modulating immune responses, aging, and fostering tissue regeneration [4–7]. In addition to the well-established significance of platelets in cardiovascular diseases, their critical involvement in the pathophysiology of cancer, inflammatory diseases, and infections has also been extensively demonstrated [3,8–11] (**Figure 1**A).

Despite their small size and anucleate status, platelets possess a diverse array of RNAs, including messenger RNAs (mRNAs), structural and catalytic RNAs, and regulatory RNAs. These RNAs play crucial roles in identifying disease biomarkers, explaining genetically or environmentally induced changes in platelet function, and facilitating the translation of mRNAs into proteins, as well as transferring RNAs to recipient cells to regulate various functional processes [12]. The study of mRNAs and microRNAs (miRNAs) has led to the emergence of “plateletomics” [13,14]. Research into human platelet miRNA–mRNA networks associated with age and gender has unveiled significant insights through integrated plateletomics, highlighting the intricate regulatory roles that these RNAs play in platelet biology.

Nowadays, there has been a surge in research focusing on platelet-related omics. This encompasses various investigative approaches, including transcriptomics, proteomics, genome-wide association study (GWAS), and transcriptome-wide association study (TWAS). Furthermore, using high-throughput sequencing and mass spectrometry technologies, researchers have undertaken significant efforts to unravel the intricate connections between genes, proteins, platelets, and diseases at various omics levels [15–18]. The primary goal of these studies is to deepen our understanding of platelets and their involvement in various diseases. By unraveling the intricacies of platelet biology, researchers aim to accelerate the development of innovative applications in diagnostics, prognostics, and therapeutics, ultimately improving patient care.

However, the retrieval, integration, and visualization of these crucial disease–gene associations, along with omics datasets, pose a formidable challenge. Valuable information is scattered across numerous scientific publications, making the process laborious and time-consuming.

Despite the existence of various resources serving different purposes, a comprehensive dedicated resource specifically for platelets is still lacking. Many of the existing resources only provide one type of omics data, limiting their scope and usefulness. Specifically, the Platelet Expression Atlas (PEA) aims to provide comprehensive platelet expression database for diseases [19]; the blood platelets-based gene expression database, PltDB, also focuses on platelet expression and offers visualization of transcriptomics profiles [20]; the human platelet repository PlateletWeb provides a novel systems biology workbench for the analysis of platelet signaling in the functional context of integrated networks [21]; Human Platelet Antigen (HPA) serves as the principal site for cataloging the current list of human platelet alloantigens. Therefore, there is an urgent need for a comprehensive and meticulously curated repository of robust disease–gene associations that encompasses a wide range of platelet-related disorders and diseases.

Here, we present PlateletBase (http://plateletbase.clinlabomics.org.cn/), a comprehensive knowledgebase specifically curated for platelet-related disorders. PlateletBase is designed for the curation, integration, visualization, and analysis of platelet and disease-related knowledge. Setting itself apart from existing databases, PlateletBase offers an extensive collection of high-quality disease–gene associations, drug–target interactions, and multi-omics datasets. This platform not only aggregates a wealth of valuable associations and interactions, meticulously curated from published literature, but also integrates a wide range of molecular profiles obtained through multi-omics data analysis. As a result, PlateletBase promises to be an invaluable resource for uncovering the molecular mechanisms driving the progression of platelet-related disorders and diseases.

## Data collection and processing

### Knowledge curation and integration

Platelet-related genomic alterations were collected from GWAS Catalog [22], TWAS Atlas [23], and International Society on Thrombosis and Haemostasis (ISTH) Gold Variants project [24]. To obtain comprehensive data on platelet antigens and antigen genes, the HPA Database and HPA Gene Database were used as sources. The information from these two databases was integrated into a single statistical platelet antigen dataset, providing systematic coding. In order to obtain pathways relevant to platelets, the latest version of the Molecular Signatures Database (MSigDB, v2023.2), containing tens of thousands of annotated gene sets for use with Gene Set Enrichment Analysis (GSEA) software, was downloaded. Keywords such as “platelet”, “thrombocyte”, and “coagulation” were utilized in the search. To assemble a collection of platelet-related ligand–receptor pairs, we initially employed the LIgand-receptor ANalysis frAmework (LIANA) R package to extract and amalgamate ligand–receptor pairs from 77 databases such as CellTalkDB, CellChatDB, CellPhoneDB, and CellCall, supported by experimental evidence and manual curation [25–27].

To obtain data on the association between platelet proteomes and diseases, extensive literature searches were conducted using the keywords “proteomics” and “platelet”. Eventually, 25 relevant studies were compiled, covering 21 different diseases with a total sample size of 1185 individuals. Referencing the book “Platelet”, three classic articles on platelet proteomics were identified, focusing on resting-state platelets, activated platelets, and platelet membrane proteins, which serve as reference data. The integration of these reference data with the compiled literature yielded a total of 4869 different types of proteins identified in platelet proteomes. For better understanding, proteins were numbered based on their expression levels in resting state, with annotations indicating their expression levels under different conditions [28–30].

To obtain data on the association between platelets, drugs, and genes, we conducted searches in three authoritative databases for drug–gene associations using multiple platelet-related keywords: The Comparative Toxicogenomics Database (CTD) [31], the large-scale bioactivity database for drug discovery (ChEMBL) [32], and the Pharmacogenomics Knowledge Base (PharmGKB) [33]. Through manual curation, we compiled 1833 entries of platelet-related drug information, involving 80 genes, 148 diseases, and 225 articles.

To coordinate disease nomenclature and definitions, terminology or identifiers (IDs) from the Disease Ontology were retrieved and mapped to the corresponding diseases [34]. In order to provide consistent names for all collected genes, gene names were unified with the help of the gene symbol–alias conversion table from the Human Genome Organisation (HUGO) Gene Nomenclature Committee (HGNC) database (2021.4.23 version) [35]. To highlight recent advancements in platelets, we conducted a search for platelet-related reviews from the past decade on PubMed and Google Scholar. We selected authoritative and impactful reviews based on citation counts and journal influence, aiming to present the latest knowledge in the field of platelets.

### Transcriptome data collection and raw data processing

The platelet transcriptome refers to the set of all RNA molecules within platelets. Although platelets are anucleate cells, they possess a rich array of RNA, including mRNA, miRNA, long non-coding RNA (lncRNA), and circular RNA (circRNA). To obtain data on the association between the platelet transcriptome and diseases, we searched public databases and relevant literature for platelet-related RNA sequencing (RNA-seq) raw data. All collected data were processed through a consistent bioinformatics pipeline. First, the quality of the raw RNA-seq FASTQ data was assessed using FastQC (v0.11.9) and MultiQC (v1.13) [36]. Based on these results, low-quality reads and adapter sequences were filtered out using fastp (v0.22.0) to obtain clean reads. Next, Spliced Transcripts Alignment to a Reference (STAR, v2.7.10b) was used to align the clean reads to the human reference genome (GRCh38), and quantification was performed using HTSeq (v2.0.2) to convert the aligned reads into count values for each gene. The expression values were then normalized to transcripts per million (TPM), and Ensembl gene IDs were converted to gene symbols, retaining the one with the highest mean expression if multiple gene IDs corresponded to the same gene symbol. This process yielded processed mRNA and lncRNA data. For circRNA detection, the clean reads were additionally aligned using Burrows-Wheeler Aligner (BWA, v0.7.17) and detected using CircRNA Identifier (CIRI, v2.0.6) [37]. Expression values were normalized to reads per million (RPM) and similarly converted to gene symbols to obtain the final circRNA data.

For mRNAs, to ensure gene generality and comparability, genes missing in more than 25% of patients were removed, while those missing in less than 25% were filled with 0. After applying log_2_ (TPM + 1) transformation, the ComBat function from the SVA R package was used to remove batch effects, considering different project sources as batch factors. The curated list of all HUGO genes was used as the standard gene names, and genes were numbered based on their expression levels from high to low in 802 normal samples, resulting in a total of 17,812 genes.

To identify differential mRNAs between each physiological or pathological state and the normal group, the Wilcoxon rank-sum test was used for each disease group compared to the normal group, with thresholds of *P* < 0.05 and |log_2_ fold change (FC)|> 0.5, identifying 8858 differentially expressed mRNAs across various disease states. To calculate characteristic mRNAs for each physiological state, we considered any given condition as the candidate state (case group) and all other groups as the control group, and we calculated specific mRNAs for different groups. Using the Wilcoxon rank-sum test with thresholds of *P* < 0.05 and |log_2_ FC| > 0.5, we identified the specific genes. For other RNAs, please refer to the corresponding sections of the website for analysis methods.

For miRNAs, we extracted the batch-corrected miRNA dataset from PltDB [20]. By ranking the miRNAs based on their expression levels in the control group, we defined a total of 8468 miRNAs. This dataset involves 167 samples and covers 6 different diseases. To identify differential miRNAs between each physiological or pathological state and the normal group, the Wilcoxon rank-sum test was used for each disease group compared to the normal group, with thresholds of *P* < 0.05 and |log_2_ FC| > 0.5.

Ultimately, our platelet transcriptome dataset covers 41 pathophysiological conditions with a total of 4408 samples. The final dataset comprises 17,926 types of mRNAs, 756 types of lncRNAs, 2493 types of circRNAs, and 8468 types of miRNAs, all expressed across various physiological and pathological states.

### Database construction and web interface implementation

PlateletBase is built using Spring Boot (https://docs.spring.io/spring-boot/index.html), MyBatis (https://mybatis.net.cn/), and MySQL (http://www.mysql.org). Its web interface is developed with CSS3, Axios, Element-UI (v2.15.8), Vue-Router (v3.0.6), and Vue (v2.x). Comprehensive statistical visualization of the data is achieved through Hypertext Markup Language (HTML) widgets, R, and Python, primarily utilizing ggplot2 (v3.5.1) and plotly (v5.19.0) (Figure 1B).

### Knowledge graph construction

To better illustrate the correlations among various omics of platelets, this analysis module incorporates the established relationships between RNA and proteins, DNA, and RNA identified in experiments or literature from the RNAInter (v4.0) dataset [38]. Additionally, we integrated expression quantitative trait locus (eQTL) data (GTEx_Analysis_v10_eQTL) to establish connections between genomic information, gene expression, and tissue localization [39]. Furthermore, we consolidated the differential expression tables of mRNAs and the proteome according to gene names to effectively demonstrate the relationship between mRNA expression and protein expression within platelets. To facilitate Chinese users, the main interface has been translated into Chinese, creating a Chinese version. Users can easily switch between Chinese and English by clicking the language toggle button.

### Analysis module construction

The analysis section, based on Python, employs various statistical methods such as Pearson, Spearman, and Kendall correlation analyses, as well as Wilcoxon rank-sum test and Fisher’s exact test. It implements a range of analytical tools and visualizes the results using the matplotlib package to create correlation plots, heatmaps, box plots, and bar charts.

## Database content and usage

PlateletBase encompasses approximately 9381 GWAS entries, 2115 TWAS entries, 897 clinical variations, 17,926 mRNA entries, 756 lncRNA entries, 2493 circRNA entries, 8468 miRNA entries, 4869 proteins, 40 platelet antigens, 65 platelet-related pathways, 2614 ligand–receptor pairs, 1833 drugs, and 97 platelet resources (Figure 1C). These entries have been meticulously curated, ensuring that each is supported by experimental evidence. PlateletBase offers a user-friendly interface tailored for efficient querying, manipulation, browsing, visualization, and analysis of intricate details regarding platelet proteins and genes. This resource promises to significantly advance research endeavors in platelet functionality and applications.

### Platelet-related genomic alterations

**Figure 2:**
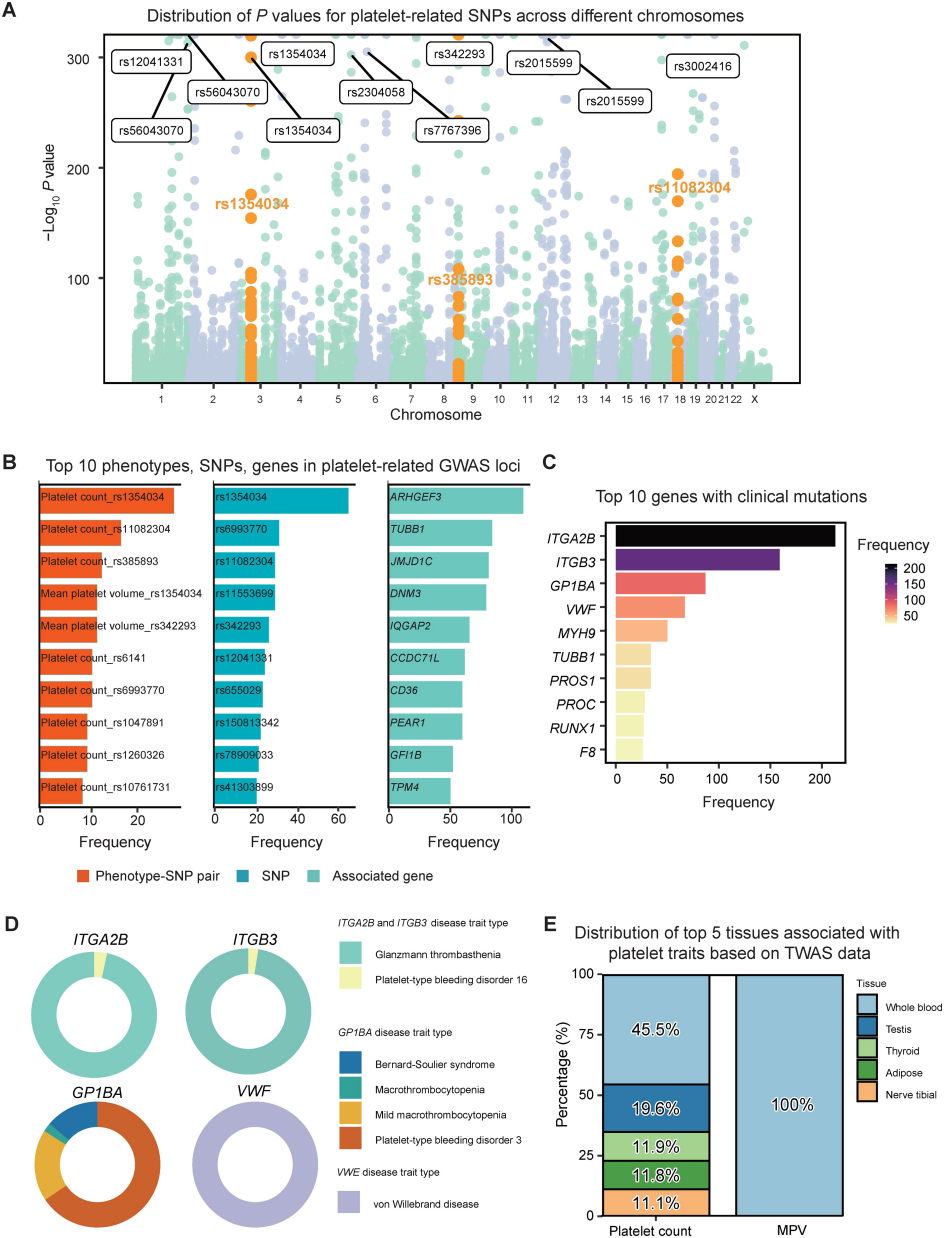
Statistics from platelet-associated genomic datasets **A**. Manhattan plot showing the relationship between chromosome position and −log_10_  *P* value for all platelet-related GWAS loci. The top 3 most frequent (phenotype or disease involved) loci are highlighted in orange, and SNPs with −log_10_  *P* value > 300 are labeled. **B**. Bar graph showing the top 10 phenotype–SNP pairs, SNPs, and genes in platelet-related GWAS loci. **C**. Bar graph showing the top 10 genes carrying clinical mutations associated with platelets. **D**. Ring diagrams showing the disease composition corresponding to the top 4 clinical gene mutations. **E**. Bar chart showing the distribution of top 5 tissues associated with platelet count and MPV based on TWAS data. MPV, mean platelet volume.

Platelet-related traits have a high genetic heritability, with up to 84% of the variance in platelet count and 75% of the variance in mean platelet volume (MPV) attributed to genetic factors [40]. Early extensive research has studied the relationships between various platelet phenotypes and genetics. The Manhattan plot in **Figure 2**A shows that these platelet phenotypes do not seem to prefer chromosomal distribution. PlateletBase integrates 112 genetically-related platelet phenotypes, such as platelet aggregation, platelet count, and MPV, among which the most important phenotypes are platelet count and MPV. The three single nucleotide polymorphism (SNP) loci most frequently linked to phenotypes are rs1354034 (28 phenotypes), rs385893 (17 phenotypes), and rs11082304 (13 phenotypes) (Figure 2A). The top 3 most frequently occurring pairs of phenotypes and SNP loci are platelet count–rs1354034, platelet count–rs11082304, and platelet count–rs385893 (Figure 2B). Studies have shown that the rs1354034 mutation can affect platelet function and be associated with ischemic stroke subtypes [40,41]. The minor allele mutation of rs11082304 is associated with platelet count [42].

Clinically relevant variations may significantly impact a patient’s life. GoldVariants, developed and tested by 30 expert centers, plays a crucial role in identifying and validating clinically relevant variations [24]. The clinically relevant variations in this database are primarily derived from the variations identified in GoldVariants. Analyses indicate that these clinical mutations predominantly arise from important platelet-related genes, such as *ITGA2B*, *ITGB3*, and *GP1BA*, which are critical for platelet function and associated with various bleeding disorders (Figure 2C).

The distribution of mutations for the four most frequently occurring clinically relevant genes is as follows: *GP1BA* mutations lead to Bernard–Soulier syndrome (13.8%), macrothrombocytopenia (2.3%), and mild macrothrombocytopenia (18.4%); *ITGA2B* and *ITGB3* mutations are associated with Glanzmann thrombasthenia (96.7% and 97.5%, respectively) and platelet-type bleeding disorder (approximately 3% each); *VWF* mutations cause von Willebrand disease (100%). These mutations affect platelet function, resulting in varying degrees of bleeding tendency (Figure 2D).

### Platelet transcriptome changes associated with diseases

While GWAS analyses have uncovered numerous common genetic variants linked to complex traits, the precise causal variants and genes at these loci frequently remain elusive, with a handful of exceptions. TWAS analyses integrate GWAS and eQTL data to forecast gene expression levels for GWAS samples and then assess for associations between the predicted expression and traits [43]. We can see that some tissues are burdened with increased levels of risk genes for given platelet traits. Eventually, 7 relevant studies were compiled. The two most frequently studied phenotypes were MPV and platelet count. Across 52 different tissues, aside from whole blood, the most frequently occurring tissues were testis, thyroid, and adipose. Further analysis revealed that the genes related to MPV were all expressed in the blood, while the genes associated with platelet count were expressed in various tissues (Figure 2E).

**Figure 3:**
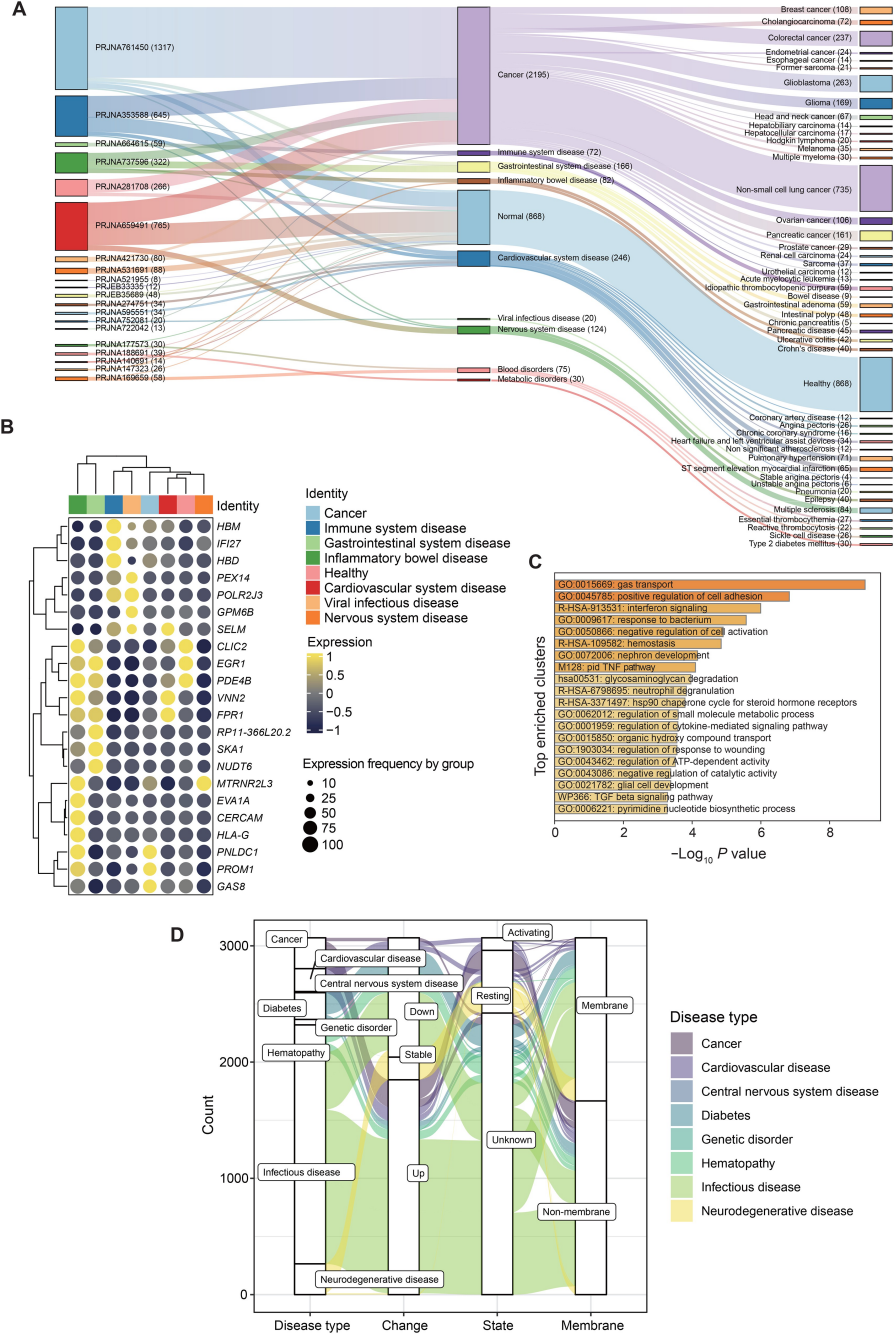
Statistics from curated platelet transcriptomic and proteomic data **A**. Sankey diagram showing the disease distribution of the transcriptomic datasets. The numbers in parentheses indicate sample size. **B**. Heatmap showing feature gene expression in platelets for several major disease types. **C**. Bar chart showing the enriched terms of top 150 genes highly expressed in platelets from healthy individuals. **D**. Sankey diagram showing the disease distribution of the proteomic data.

Research indicates that platelet mRNAs can be translated into proteins or transferred to recipient cells, thereby regulating functional processes. This database extracts 20 sets of original data, 3711 samples, and 41 different conditions from National Center for Biotechnology Information (NCBI) (**Figure 3**A).

In order to better demonstrate the disease-specific expression of platelet-related RNAs, we extracted the three genes with the greatest differential expression for each major disease category, and displayed them in a heatmap. The inherent similarities and differences between diseases can be well reflected by the differential platelet expression profiles. The tumor diseases form a distinct cluster, while the viral infections and immune system diseases form a similar branch (Figure 3B). We enriched the top 150 genes highly expressed in the healthy group through Metascape, and found normal cell functions such as gas transport and positive regulation of cell adhesion [44] (Figure 3C).

### Platelet proteome changes associated with diseases

The composition, localization, and activity of proteins are essential for platelet function and regulation. The current advancements in mass spectrometry-based proteomics offer significant potential to detect and quantify thousands of proteins from minimal sample quantities, uncover various post-translational modifications, and track platelet activity in response to drug treatments. This database extracts data from 25 publications covering 21 different diseases, identifying 1345 differentially expressed proteins. Sankey diagram analysis shows that the majority of highly expressed proteins in cancers and cardiovascular diseases are primarily derived from activated platelets (Figure 3D).

### Platelet-related antigens

Alloantibodies against HPA are implicated in several immune-mediated platelet disorders, such as fetal and neonatal alloimmune thrombocytopenia and platelet transfusion refractoriness. The detection and identification of these HPA alloantibodies are critical for accurate diagnosis and the implementation of appropriate patient care strategies. In PlateletBase, 40 distinct platelet antigens are compiled, with 32 located on chromosome 17, as referenced in 54 publications.

### Platelet-related molecular signatures and manually curated gene sets

**Figure 4:**
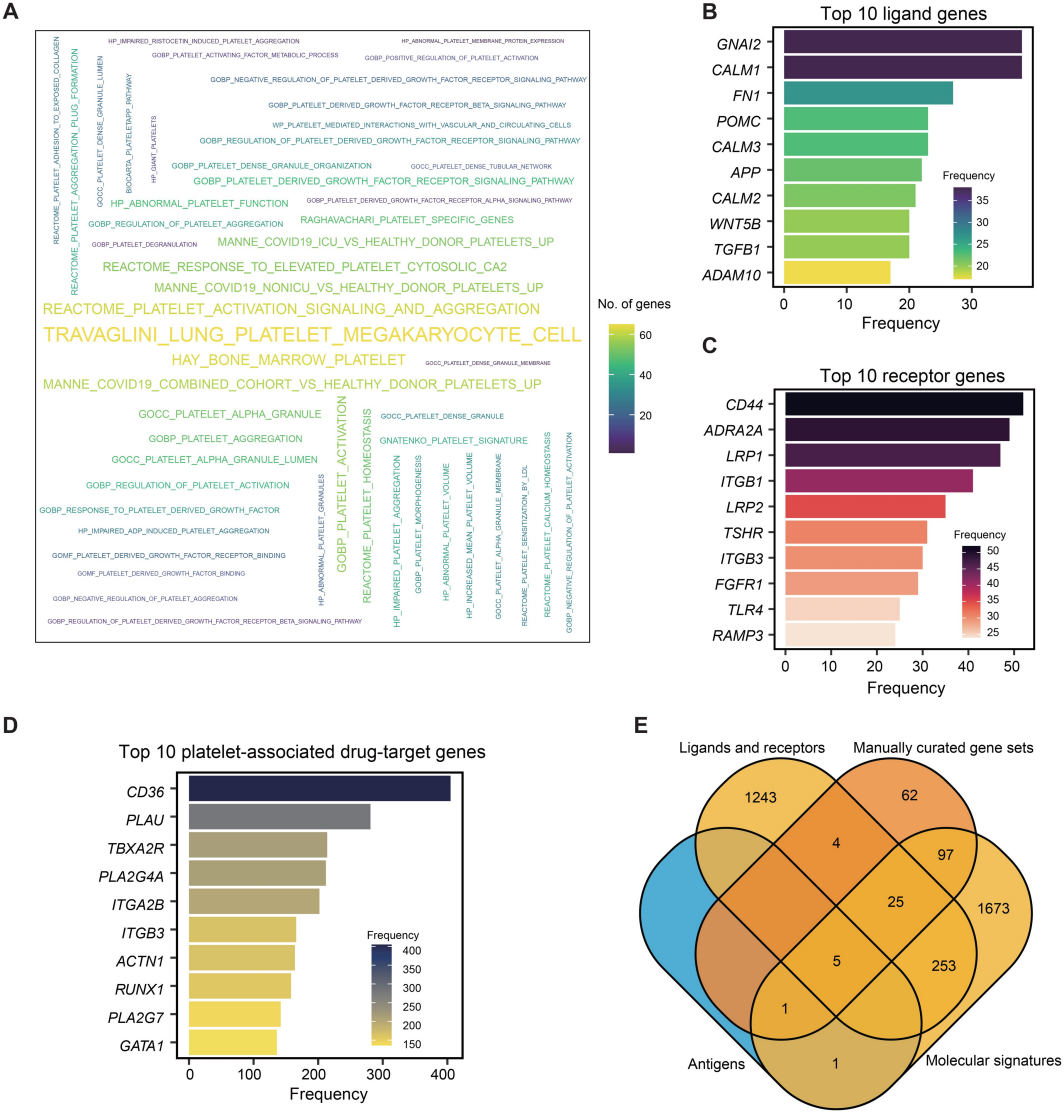
Statistics from omics knowledge and drugs associated with platelets **A**. Word cloud displaying all platelet-related molecular signaling pathways in the database. The font size and color are proportional to the rank of the pathways and the number of genes in each pathway, respectively. **B**. Bar chart showing the top 10 most frequent ligand genes. **C**. Bar chart showing the top 10 most frequent receptor genes. **D**. Bar chart showing the top 10 most frequent platelet-associated drug-target genes. **E**. Venn diagram showing gene overlap.

MSigDB is a comprehensive resource that houses a collection of gene sets representing specific biological pathways, molecular processes, and cellular functions. These gene sets are curated from diverse sources, including experimental studies, computational predictions, and literature annotations. The database facilitates the systematic exploration of gene expression data by allowing researchers to analyze and interpret their results in the context of known biological pathways and signatures. Ultimately, 65 platelet-related molecular signatures are compiled, involving 2056 genes, which are displayed by word cloud (**Figure 4**A).

To provide more molecular biology information for platelet research, we compiled 15 datasets related to platelet production, adhesion, reticulated platelets, and other relevant areas, involving 194 genes or proteins. Additionally, using a method similar to the MSigDB, we sorted 150 (or fewer if the criteria are not met) highly or lowly expressed genes by log_2_ FC, ensuring *P* < 0.05 in platelet mRNA transcriptome data. Collectively, we compiled 82 gene sets associated with 41 disease states, based on differentially expressed genes compared to normal platelets. Furthermore, we compiled disease-specific gene sets by comparing various diseases and other conditions, resulting in 84 gene sets across 42 conditions, including normal states.

### Platelet-related ligands and receptors

Platelet receptors play crucial intercellular communication roles by serving as both ligands and receptors, facilitating interactions between platelets and other cells or extracellular molecules. These receptors mediate signaling pathways that regulate platelet activation, adhesion, and aggregation in response to external stimuli. By recognizing and binding to specific ligands, such as adhesive proteins or agonists, platelet receptors initiate cellular responses that contribute to hemostasis, thrombosis, and other physiological processes. Through these intercellular communication mechanisms, platelet receptors coordinate complex cellular interactions within the vascular system. In PlateletBase, we compiled a comprehensive set of 2614 ligand–receptor pairs, encompassing 770 receptors and 808 ligands. Among these, 77 pairs have been previously reported, while 1528 receptor genes and 1009 ligand genes expressed in platelets were novel discoveries. Top 10 genes are displayed by bar plots (Figure 4B and C).

### Platelet-related drug–target interactions

Understanding medications for platelet-related diseases is crucial for effective management. These drugs, such as antiplatelet agents and anticoagulants, play a vital role in preventing thrombosis and managing cardiovascular conditions. Genetic factors can influence how individuals respond to these medications, highlighting the importance of personalized treatment approaches. However, emphasizing the significance of platelet-related medications underscores their direct impact on patient care and clinical outcomes. Through manual curation, we compiled 1833 entries of platelet-related drug information, involving 80 genes, 148 diseases, and 225 publications. *CD36* is the highest frequency gene, which encodes platelet glycoprotein IV and acts as both a signaling receptor and a transporter for long-chain fatty acids [45] (Figure 4D). We further applied Venn diagram analysis to display the gene overlap among the different omics data. We found that the gene sets for “platelet-related ligands and receptors” and “platelet-related molecular signatures” shared the largest number of common genes (Figure 4E).

### Personalized analysis module for platelet omics

This database has detailed help documentation. It provides a simple introduction to the navigation bar, where the standout feature is the analysis module, which facilitates customized analysis by researchers to obtain more information. Compared to previous platelet-related databases, our database is the first platelet multi-omics database, and the sample size of various omics data exceeds that of previous databases, with more disease types covered (**Table 1**). Based on the transcriptome data in the database and backend programs, this database provides five analysis and visualization methods: “similar gene analysis by correlation” allows users to select a correlation method, disease types, gene of interest, and result proportion to see genes most correlated with the input gene; “pair correlation” enables users to analyze the correlation between two genes in selected diseases, with results shown in a scatter plot; “multi-correlation” lets users examine correlations among multiple genes, with results in a correlation heatmap; “expression rank in all conditions” allows users to view a gene’s expression across diseases and healthy samples in a box plot; and “clinical analysis” enables users to explore a gene’s expression under different clinical conditions within selected diseases, presented in a box plot. Users can choose the RNA type (mRNA, lncRNA, circRNA, and miRNA) and the appropriate analysis method to get the corresponding results.

**Table 1:** Comparison of PlateletBase with existing platelet-related databases

Name	Omics type (sample size/dataset count)	Multi-omics	Drug list	Knowledge graph	Platelet function annotation	Analysis module
PEA	Transcriptomics (1260 samples)	No	No	No	No	No
PltDB	Transcriptomics (2408 samples)	No	No	No	No	Yes
PlateletWeb	Proteomics (26 datasets,)	No	No	No	No	No
HPA	Human platelet alloantigens	No	No	No	No	No
GoldVariants	Clinical mutations	No	No	No	No	No
PlateletBase	Genomics / transcriptomics (3711 samples) / proteomics (1185 samples) / knowledge annotations	Yes	Yes	Yes	Yes	Yes

*Note*: PEA, Platelet Expression Atlas; HPA, Human Platelet Antigen.

The users also can conduct enrichment analysis. This function is designed to analyze whether specific genes or molecules are significantly enriched in certain biological contexts, helping users quickly identify potential key biological mechanisms or pathways. Users can input a gene list of interest and select relevant gene sets from the database based on their research needs (with the option to select all). Enrichment analysis is then performed using Fisher’s exact test, and the results are presented in a tabular format, including the following information: gene set name, the number of genes in the gene set, the number of overlapping genes, the proportion of overlapping genes, *P* value, and adjusted *P* value. Additionally, a bar chart visualizing the top 10 most significantly enriched gene sets is generated for better interpretation.

### Integrate multi-omics information through knowledge graph

Considering the pertinent isolation of multi-omics data, it is imperative to establish connections among various data types. This section is organized into three principal modules. The first module employs RNA as the initial point for interactions. Users may select from three distinct interaction modalities: RNA–RNA, RNA–DNA, and RNA–protein. Upon entering a gene name, users can obtain both graphical and tabular representations illustrating the gene’s involvement in network interactions across different forms. The second module is centered on SNP loci, aiming to elucidate the functional associations between SNPs correlated with platelet phenotypes and gene expression within specific tissues. Users can input a gene, SNP locus, or tissue name to generate corresponding network relationship diagrams and tables. The final module integrates tables of platelet RNA and platelet proteins, utilizing gene symbols as linking entities. This facilitates users in exploring the associations between the same gene in both RNA and protein forms and their relevance to various diseases.

### Case study 1: gray platelet syndrome

**Figure 5:**
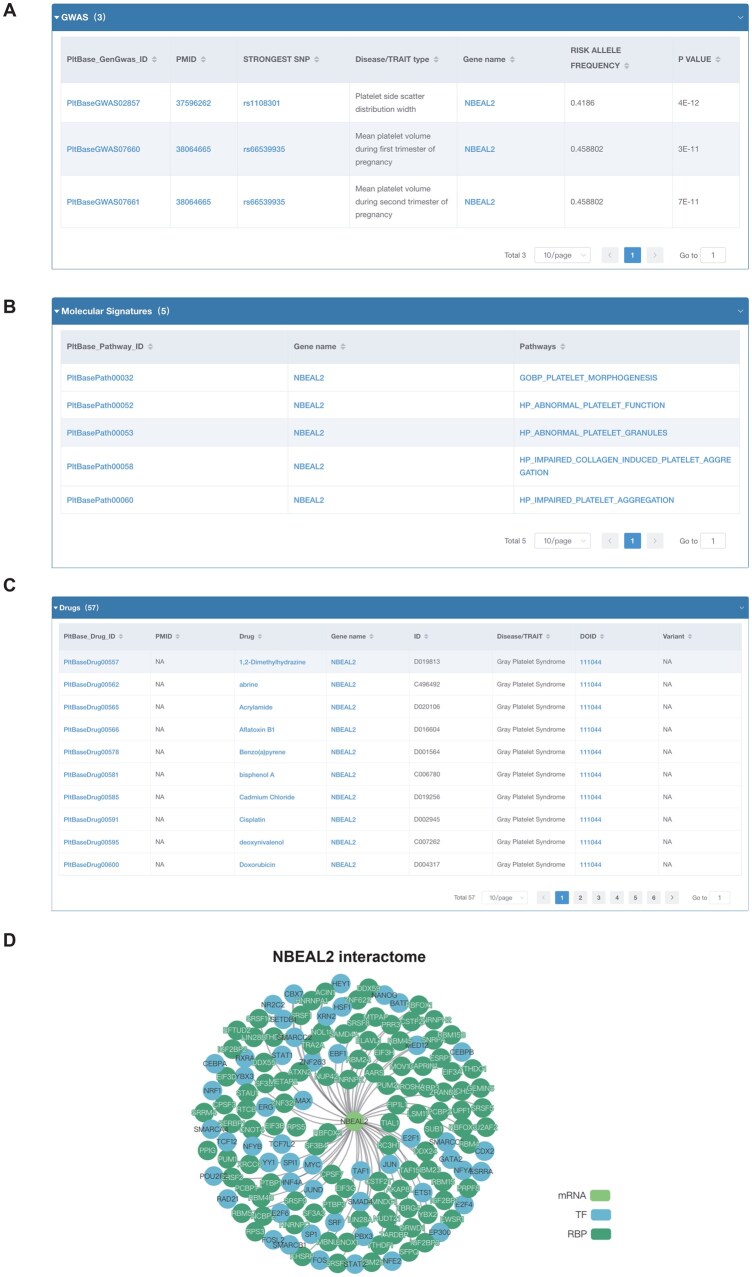
Case report of gray platelet syndrome **A**. *NBEAL2*-related GWAS locus information. **B**. Molecular pathway information associated with *NBEAL2*. **C**. List of drugs related to *NBEAL2*. **D**. Protein network diagram related to NBEAL2. TF, transcription factor; RBP, RNA-binding protein.

Previous studies have indicated that gray platelet syndrome (GPS) is a rare platelet disorder characterized by abnormalities in α-granules and mutations in *NBEAL2*, featuring macrothrombocytopenia and a specific deficiency of α-granules and their associated contents [46]. A user seeking to understand GPS can first navigate to the genomics section and click on the clinical variant subsection, selecting GPS. This will reveal 16 distinct mutations associated with the pathogenesis of GPS. Consistent with prior research, all identified mutations involve the *NBEAL2* gene. By accessing the hyperlink for this gene, users can explore more in-depth studies. GWAS data indicate that the SNP loci of the *NBEAL2* gene are correlated with MPV characteristics, aligning with the phenotypic features of GPS (**Figure 5**A). In the omics knowledge section, *NBEAL2* is associated with α-granules, appearing within several platelet-related gene sets (Figure 5B).

In the drugs section, multiple therapeutic agents targeting the *NBEAL2* gene for the treatment of GPS are listed (Figure 5C). Furthermore, transcriptomic and proteomic analyses and knowledge graph related to *NBEAL2* (Figure 5D) have revealed additional relationships between *NBEAL2* and various diseases and proteins, potentially offering new avenues for mechanistic studies or repurposing existing medications.

### Case study 2: angina pectoris

Angina significantly contributes to global morbidity, with its assessment being variable and influenced by multiple factors. Cardiac troponins have replaced other blood biomarkers for diagnosing myocardial injury [47]. However, unstable angina is less frequently diagnosed with high-sensitivity cardiac troponin (hs-cTn) assays [48]. This study aims to identify platelet-based diagnostic biomarkers for angina using this database. Initially, we accessed the transcriptomics section to identify differentially expressed genes associated with angina compared to healthy individuals, as well as specific differentially expressed genes compared to non-angina patients, sorting them by log_2_ FC. We selected the gene with the highest log_2_ FC, identifying *CAPN10* as a candidate biomarker.

**Figure 6:**
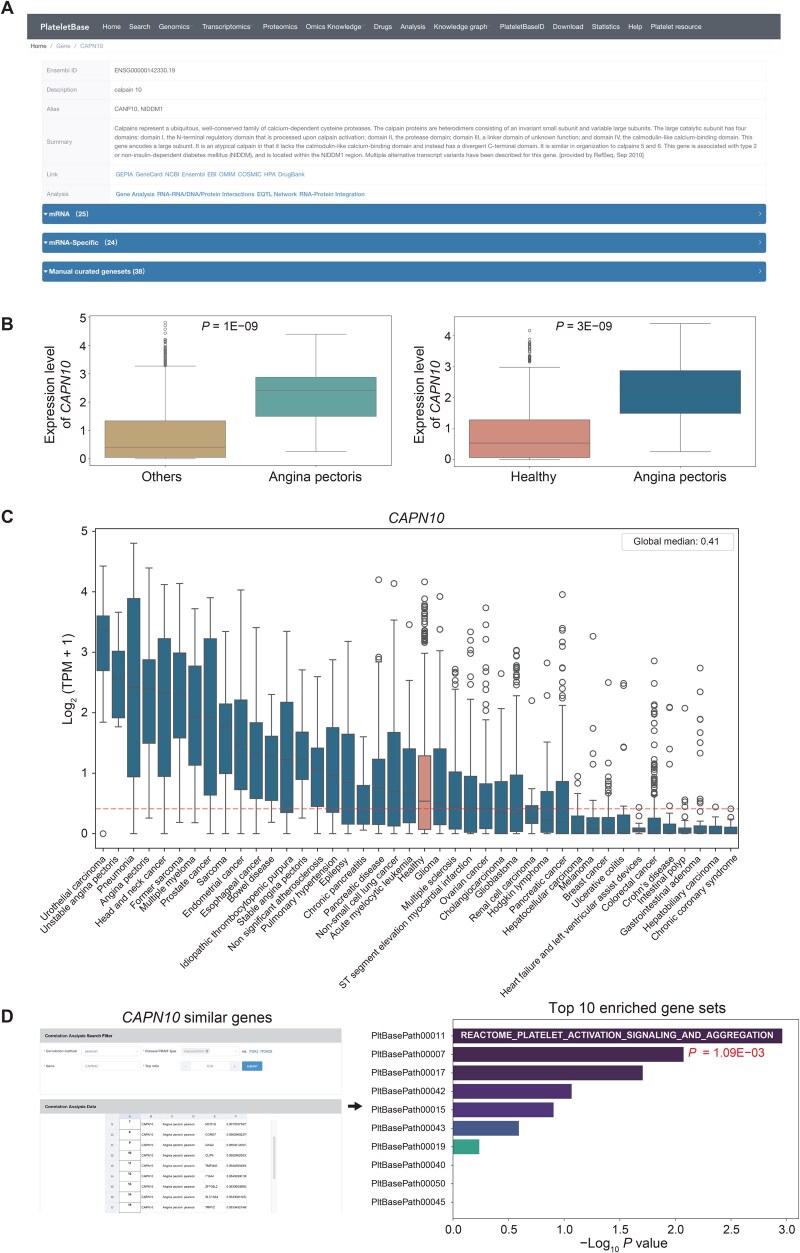
Case report of angina pectoris **A**. Basic information about the differentially expressed gene *CAPN10* related to angina pectoris. **B**. Box plots showing the expression levels of *CAPN10* in angina pectoris *vs.* “others” (left) and in angina pectoris *vs.* healthy individuals (right). “Others” indicates all patients with other disease states. **C**. *CAPN10* expression rank across all conditions. **D**. The top 100 genes exhibiting high expression-based correlation with *CAPN10* at the same biological level were identified. These genes were found to be enriched in platelet-related molecular signaling pathways through enrichment analysis and visualized using the plot function in PlateletBase. TPM, transcripts per million.

We examined the basic information for this gene through its hyperlink (**Figure 6**A) and confirmed the validity of the differential expression using the built-in plotting tool (Figure 6B). To consider *CAPN10* as a biomarker, we needed to identify potential differential diagnosis factors; therefore, we ranked diseases based on *CAPN10* expression. We found that patients with unstable angina exhibited the highest platelet expression of *CAPN10* among over 40 diseases, necessitating differentiation from only a few conditions, such as urothelial carcinoma (Figure 6C).

To further investigate the potential function of this gene in platelets, we utilized the similar gene module to obtain the top 100 similar genes and employed the enrichment module, revealing that *CAPN10*-related genes are primarily enriched in gene sets associated with platelet activation (Figure 6D). Therefore, this tool can be utilized for the exploration of diagnostic biomarkers.

## Discussion and perspectives

In this study, we present PlateletBase, a comprehensive knowledge base that provides a high-quality collection of data on human platelets, including associated diseases, genes, drugs, and omics profiles. Based on integration from published literature and related databases, the current version of PlateletBase contains 10,278 genomic entries, 31,758 transcriptomic entries, 4869 proteomic entries, 2614 omics knowledge entries, 1833 drugs, 97 platelet resources, 438 diseases/traits, and 6 analysis modules. Future directions include: (1) frequent curation and expansion of all data sections; (2) integration and analysis of additional multi-omics datasets for platelets; and (3) improvement of the web interface and development of tools to facilitate multi-omics data mining and visualization. It is important to note that many aspects of this study are based on data mining, which necessitates further validation to corroborate the findings. We call on the global scientific community to collaborate in building PlateletBase into a valuable resource covering more comprehensive associations, interactions, and omics data, in order to further advance high-quality, curated knowledge for platelet and disease research.

## Data Availability

PlateletBase is freely available online at http://plateletbase.clinlabomics.org.cn/ and does not require user registration. PlateletBase has been submitted to Database Commons [49] at the National Genomics Data Center (NGDC), China National Center for Bioinformation (CNCB), which is publicly accessible at https://ngdc.cncb.ac.cn/databasecommons/database/id/9704.

## CRediT author statement

**Huaichao Luo:** Methodology, Data curation, Software, Visualization, Writing – original draft, Funding acquisition. **Changchun Wu:** Software, Supervision. **Sisi Yu:** Resources. **Hanxiao Ren:** Resources. **Xing Yin:** Resources. **Ruiling Zu:** Resources, Funding acquisition. **Lubei Rao:** Resources, Funding acquisition. **Peiying Zhang:** Resources. **Xingmei Zhang:** Resources. **Ruohao Wu:** Resources. **Ping Leng:** Resources, Supervision. **Kaijiong Zhang:** Resources, Supervision. **Qi Peng:** Resources, Supervision. **Bangrong Cao:** Conceptualization, Supervision. **Rui Qin:** Software, Supervision. **Hulin Wei:** Methodology, Data curation, Software, Visualization. **Jianlin Qiao:** Conceptualization, Supervision. **Shanling Xu:** Conceptualization, Supervision, Funding acquisition. **Qun Yi:** Conceptualization, Supervision, Funding acquisition. **Yang Zhang:** Resources, Supervision. **Jian Huang:** Conceptualization, Supervision. **Dongsheng Wang:** Writing – review & editing, Funding acquisition. All authors have read and approved the final manuscript.

## ORCID

0000-0002-8632-5230 (Huaichao Luo)

0009-0003-9864-2756 (Changchun Wu)

0009-0002-4728-0241 (Sisi Yu)

0009-0000-9003-9432 (Hanxiao Ren)

0009-0006-2014-5794 (Xing Yin)

0000-0002-9803-1459 (Ruiling Zu)

0000-0002-8243-0076 (Lubei Rao)

0009-0008-7805-8380 (Peiying Zhang)

0000-0002-6047-0640 (Xingmei Zhang)

0009-0007-3130-3425 (Ruohao Wu)

0000-0002-4019-2153 (Ping Leng)

0000-0002-5459-0144 (Kaijiong Zhang)

0009-0002-0452-6985 (Qi Peng)

0000-0002-3865-3563 (Bangrong Cao)

0009-0003-3573-2789 (Rui Qin)

0009-0001-7099-8174 (Hulin Wei)

0000-0002-0456-0940 (Jianlin Qiao)

0000-0002-1801-1440 (Shanling Xu)

0009-0007-2136-0354 (Qun Yi)

0000-0002-1317-120X (Yang Zhang)

0000-0003-3282-8892 (Jian Huang)

0000-0003-1241-4908 (Dongsheng Wang)
